# The spontaneous resolution of heavy menstrual bleeding in the perimenopausal years

**DOI:** 10.1111/j.1471-0528.2012.03282.x

**Published:** 2012-02-08

**Authors:** M Shapley, M Blagojevic, KP Jordan, PR Croft

**Affiliations:** Primary Care Sciences Research Centre, Keele UniversityStaffordshire, UK

**Keywords:** Epidemiology, heavy menstrual bleeding, menopause, menorrhagia

## Abstract

**Objective:**

To obtain estimates of the rate of spontaneous resolution of heavy menstrual bleeding and to explore any association with specific menstrual symptoms.

**Design:**

Two-year prospective cohort study.

**Setting:**

Seven general practices, with 67 100 registered patients.

**Population:**

All women aged 40–54 years on the practices age–sex registers.

**Methods:**

Baseline postal questionnaire, with follow-up questionnaires sent to naturally menstruating respondents at 6, 12, 18 and 24 months.

**Main outcome measures:**

Rate of spontaneous resolution of heavy menstrual bleeding in naturally menstruating women.

**Results:**

A total of 7121 baseline questionnaires were sent out, with an initial response rate of 63%. We recruited 2051 naturally menstruating women for the prospective cohort study. The spontaneous rate of resolution of heavy menstrual bleeding varied from 8.1% (95% CI 5.3–12%) in women aged 45–49 years, who had resolution without recurrence for 24 months, to 35% (95% CI 30–41%) in women aged 50–54 years, who had resolution without recurrence for 6 months. Rates were lower in those who reported interference with life from heavy menstrual bleeding. There was a strong association between the spontaneous resolution of heavy menstrual bleeding and skipped periods in women aged over 45 years. The association with ‘cycle too variable to say’ was significant, but weaker.

**Conclusion:**

There is a high prevalence, incidence and significant spontaneous rate of resolution of heavy menstrual bleeding in naturally menstruating women during the perimenopausal years. The rates have potential use for individual women, clinical decisions, devising and implementing interventions and planning the care of populations.

*Please cite this paper as:* Shapley M, Blagojevic M, Jordan K, Croft P. The spontaneous resolution of heavy menstrual bleeding in the perimenopausal years. BJOG 2012;119:545–553.

## Introduction

Heavy menstrual bleeding is a common symptom in the years before the onset of the menopause,^1^–^4^ and a substantial number of women consult primary care with this complaint.^5^ The reasons for consultation vary from a need to understand the change in menstruation to the interference that the menstrual disturbance has on the woman’s life.^6^–^9^ Treatment options include advice, drug therapy and surgery.^10^ The National Institute of Health and Clinical Excellence have emphasised the need to favour a patient-centred psychosocial model of care, rather than a biomedical one.^10^,^11^ In order to allow women to make an informed decision regarding therapeutic options, an understanding of the likelihood of spontaneous resolution of the presenting problem without treatment is needed. This is particularly relevant to women in the perimenopausal years when the onset of the menopause will cause menstruation to cease. Trials on drugs for the treatment of menorrhagia with placebo or no treatment arms exist, but their usefulness in this context is limited by their time frame (six cycles or fewer), age group and use of secondary care populations.^10^

The mean age of the menopause in developed countries is 51 years,^12^ and race,^13^ socio-economic class,^14^ body mass index,^15^ and smoking status^16^,^17^ appear to affect the age of the menopause by up to 2 years, depending on the factors involved.^14^ It has been postulated that 60 days or more of amenorrhoea in the previous year and ‘cycles too variable to report a usual length’ in menstruating women aged 44 years and over are predictive of the menopause.^18^ Although these studies have defined the age of the menopause and identified risk factors for an earlier or delayed occurrence, their clinical usefulness has been limited, as they have not given an estimate of the chance that a woman at a particular age will cease menstruation in the next year. Only one study using menstrual charts in 393 North American women who had a natural menopause has provided annual age-related estimates of this risk.^19^ No studies have given an estimate of the chance of the spontaneous resolution of heavy menstrual bleeding.

The specific objective of this study was to obtain estimates of the prevalence and incidence of heavy menstrual bleeding in women during the perimenopausal years, and to determine the likelihood that the symptom will resolve spontaneously without recurrence over the next 6–24 months. The purpose is to aid decision-making in the community and primary care about the need for treatment for heavy menstrual bleeding in the perimenopausal years.

## Methods

The study took place in North Staffordshire, UK. The sampling frame was provided by the registered populations of seven general practices that form part of a local research network. The practices were selected to cover a mix of urban and rural areas, and affluence. The total registered population was 67 100, and the target population for the study was all women on the registers aged 40–54 years. As almost all people in the UK are registered with a general practice, regardless of whether they seek health care or not, such registers provide representative samples of the population of a local area. The inclusion criteria were women aged 40–54 years in the community who were willing and able to give consent and return a questionnaire.

The study was conducted in two phases: first, a baseline cross-sectional postal survey of the target population; and second, a prospective cohort study of naturally menstruating women identified in the baseline survey, and who consented to follow-up questionnaires at 6, 12, 18 and 24 months. At baseline and each follow-up time point women who did not return the questionnaire were sent a reminder postcard after 2 weeks, and a second copy of the questionnaire after a further 2 weeks. The baseline questionnaire was sent out on 5 September 2007.

The sample size was based on the primary outcome of resolution of heavy menstrual bleeding without recurrence for a year (heavy menstrual bleeding at baseline and no heavy menstrual bleeding at both 6 and 12 months). The estimated prevalence and resolution was derived from an earlier study of 1513 women and a 95% confidence interval, with a maximum acceptable difference of 3%.^4^

Table 1 lists the definitions of terms related to menstrual loss used in the study, derived from the questionnaire. Naturally menstruating women were defined as those who replied yes to the question ‘have you had a period in the last 6 months’ and no to questions concerning the use of female hormones, effective treatments for heavy periods (non-steroidal anti-inflammatory drugs, Tranexamic acid),^10^ an intrauterine device, pregnancy or a gynaecological operation in the last 6 months, or ever having had an endometrial ablation.

**Table 1:** Questions used in the survey and terms used in the text

Questions	Response	Term
‘Have you had a period in the last 6 months?’	‘Yes’	Menstruating
Has not in the last 6 months used female hormones, treatment of known efficacy for heavy and/or irregular periods, an intrauterine contraceptive device, been pregnant, had a gynaecological operation or ever had an endometrial ablation or hysterectomy.		Natural
‘Over the last 6 months how do you regard your periods?’	‘Fairly heavy’, ‘very heavy’ or ‘variable’	Heavy menstrual bleeding
‘Over the last 6 months has the heaviness of your periods interfered with your life?’	‘Yes’	Heaviness that interferes with life
‘Over the last 6 months have you missed or skipped a period?’	‘Yes’	Skipped period
‘Over the last 6 months what is the usual time from the start of one period to the start of the next?’	‘Too variable to say’	Cycle too variable to say
‘Have you consulted a doctor and/or nurse during the last 6 months about the heaviness of your periods?’	‘Yes’	Consulted

The instrument used in the questionnaires to determine the women’s perception of the heaviness of menstruation has been validated,^3^ and has been used in other studies by members of the study team.^4^,^8^,^9^ Heavy menstrual bleeding is defined as ‘heavy’ menstruation, which corresponds with a response to the question ‘over the past 6 months how do you regard your periods?’ of ‘fairly heavy’, ‘very heavy’ or ‘variable’. The alternative response of ‘very light’, ‘fairly light’ or ‘neither heavy nor light’ defined the term ‘non-heavy’ menstruation. A change in status from ‘non-heavy’ menstruation to ‘heavy’ menstruation was used to determine incidence. Cumulative incidence was defined as at least one new episode of ‘heavy’ menstruation during the time frame used. A change in status from ‘heavy’ menstruation to ‘non-heavy’ menstruation was used to determine resolution, whereas ‘without recurrence’ was defined as resolution for the whole time frame used.

The questionnaires contained items asking if the woman had ‘missed or skipped’ a period in the last 6 months, and a question asking her to categorise the usual time from the start of one period to the beginning of the next as <21, 21–35 days, more than 35 days or ‘too variable to say’. Other questions investigated, in the previous 6 months, other vaginal bleeding symptoms, use of treatments for heavy periods and other menstrual disturbances, consultation with primary and secondary care and, in the baseline questionnaire, factors known to influence the age of onset of the menopause (weight, height, race, current smoking status and work status).^19^ The questionnaires were piloted for comprehension and completion prior to the study.

Respondents who only partially completed the questionnaires were sent a photocopy of the missing items for them to complete. Respondents who consented to follow-up and changed their registered practice during the study period were traced using the National Health Service’s Strategic Tracing Service.

### Analysis

The cohort of women who were identified from the baseline questionnaires as naturally menstruating provided the sample for analysis. Three outcomes were the focus of attention in the analyses of these women: heavy menstrual bleeding, resolution of heavy menstrual bleeding and use of health care (consultation with a doctor and/or nurse about the heaviness of their periods). A change in status from ‘natural’ (i.e. no longer fulfilling the definition of ‘natural’ menstruation given in Table 1) at a follow-up time point excluded the individual from the cohort for the whole of the time frame, with the exception of the analyses of consultation rates, in which the woman only needed to be menstruating naturally in the first questionnaire. Loss to follow-up similarly excluded the woman from the cohort.

Age was defined separately in each of the baseline and 12-month questionnaires (‘age-defining’ questionnaires). Any one woman could contribute to more than one age-specific calculation if she contributed relevant person-time at risk during the course of the follow-up study and moved up to the next age band (annual or 5-year age bands). A woman could only contribute once to the ‘overall’ 15-year age band, and only once to a 2-year analysis, as a woman could not move up an age band.

Questionnaires were sent out at 6-monthly intervals with questions related to the presence or absence of symptoms over the past 6 months.

### Prevalence

At baseline, the prevalence of heavy menstrual bleeding and other symptoms in the past 6 months were calculated from responses to the baseline questionnaire only, and were expressed as the proportion of women reporting the symptom from all of those in the age band.

### Incidence

Cumulative incidence was defined as a change from no heavy menstrual bleeding in the age-defining questionnaire to heavy menstrual bleeding in at least one of the questionnaires during the analysis time frame. Rates of cumulative incidence for 6 months (new symptom reported in the following questionnaire), 1 year (new symptom reported in either of the following two questionnaires) and 2 years (new symptoms reported in 6-, 12-, 18- or 24-month questionnaires) were determined. For example, if a woman without heavy menstrual bleeding at baseline first reported heavy menstrual bleeding at 12 months, she would be included as an incident case for the 1- and 2-year analyses for her baseline age band, but not for the 6-month analysis. Incidences of heavy menstrual bleeding at each age were calculated in relation to the relevant denominator for that age.

Women contributed to the denominator population (‘women at risk of heavy menstrual bleeding’) for each time period of 6 months, where they started in the ‘age-defining’ questionnaire with no heavy menstrual bleeding, and their status at the end of each 6 months was dichotomised into ‘heavy menstrual bleeding’ yes or no. Separate cumulative incidence figures were calculated for 6 months, 1 and 2 years by combining each individual woman’s relevant 6-month section, so long as they remained in the cohort for the relevant duration of time.

For heaviness of menstrual bleeding that interferes with life, the cumulative incidence was defined as at least one report of both heavy menstrual bleeding and heaviness of bleeding that interferes with life on a questionnaire within the relevant time frame.

### Resolution without recurrence

Resolution was defined as a change from heavy menstrual bleeding in the age-defining questionnaire to no heavy menstrual bleeding for the whole of the analysis time frame (i.e. without recurrence). Rates of resolution for 6 months (no symptoms reported in the following questionnaire), 1 year (no symptoms reported in both the following two questionnaires) and 2 years (no symptoms reported in the 6-, 12-, 18- and 24-month questionnaires) were determined. For a woman with heavy menstrual bleeding at baseline, reports of no such symptoms at each of 6, 12, 18 and 24 months would therefore be required to be defined as resolved without recurrence for 2 years.

Women contributed to the denominator population (‘women at risk of resolution of heavy menstrual bleeding’) for each time period of 6 months if they started in the ‘age-defining’ questionnaire with heavy menstrual bleeding, and their status at the end of each 6 months was dichotomised into ‘resolution of heavy menstrual bleeding’ yes or no. Separate resolution figures were calculated for 6 months, and 1 and 2 years, by combining each individual woman’s relevant 6-month section, so long as they remained in the cohort for the relevant duration of time.

The analyses for resolution were also repeated, stratified separately for each of the characteristics of interference with life, skipped periods and ‘cycle too variable to say’, and with status defined by responses in the age-defining questionnaires. Rate ratios were calculated by dividing rate of resolution in those with a particular characteristic by the rate in those without the characteristic.

### Health-care use

The annual cumulative incidence of consultation with a doctor and/or nurse for heaviness of periods was also determined. The use of the given time frames (6, 12 and 24 months) is supported by an analysis of optimal sampling strategies concerning menstrual function.^20^ The definitions of cumulative incidence and resolution were felt to represent the information requirements of women and clinicians during a consultation, and provides a variety of rates that may be applied to individuals to meet their own personal values and needs.

Analyses were performed using SPSS 18 (IBM, New York, USA), and confidence intervals for all relevant rates were calculated using Confidence Interval Analysis.^21^ Chi-square tests were used to test differences between proportions (including trend by age), and we report *P* values (a value of <0.05 is assumed to indicate statistical significance).

## Results

### Baseline survey

A total of 7121 questionnaires were sent out at baseline; 2394 women did not return the questionnaire for no given reason. Of the 4727 returned questionnaires, 184 were returned uncompleted. Eighty-three women of these 184 had left their registered practice and could not be traced, or mail was returned as undelivered mail, moved house or addressee unknown, and 101 women refused to participate for various reasons (e.g. questions too personal or lack of time). A further 88 women who returned completed questionnaires were also excluded, as 40 reported that they had had a hysterectomy, four reported learning difficulties or mental disability and 44 were outside the age range of 40–54 years at the time of response, or it was not possible to determine the age of the participant from the questionnaire. This resulted in 4455 eligible returned questionnaires, giving a response rate of 63%.

The mean age of the resulting 4455 eligible respondents at baseline was 47 years (standard deviation 4.3 years). Non-respondents were slightly younger than respondents, with a mean age of 46 years (standard deviation 4.3 years). A total of 2949 women were menstruating (66%), of whom 2167 (73% of those menstruating) were menstruating naturally.

### Prevalence

The prevalence of heavy menstrual bleeding as a proportion of naturally menstruating women at baseline was 65% (95% CI 63–67%), with no significant variation with age (40–44 years 66%, 45–49 years 65% and 50–54 years 62%; *P* = 0.385). The prevalence of heaviness that interferes with life at baseline was 26% (95% CI 24–27%), with no significant variation with age (40–44 years 25%, 45–49 years 27%, 50–54 years 25%; *P* = 0.570). The baseline prevalence of other symptoms in naturally menstruating women was 12% (95% CI 10–13%) for ‘cycle too variable to say’ and 26% (95% CI 24–28%) for skipped period, with both symptoms showing a sharp significant age-related increase (*P* < 0.001) from 6 to 9%, respectively, in women aged 40–44 years, and 13 and 28%, respectively, in women aged 45–49 years, to 24% and 62%, respectively, in women aged 50–54 years.

### Prospective cohort study

Of the 2167 who were naturally menstruating, 116 were not registered with consent to receive further questionnaires, and thus 2051 eligible women were sent 6-, 12-, 18- and 24-month questionnaires. At the 6-month mailing, 315 did not respond and 15 were excluded because of patient withdrawal, incorrect postal address or questionnaire returned too late. The response rate was 85%. The corresponding figures for the 12-, 18- and 24-month mailings were: 346 non-responders, 15 excluded, 83% response rate; 395 non-responders, 15 excluded, 81% response rate; and 363 non-responders, 27 excluded, 82% response rate, respectively.

### Incidence

The overall (i.e. age 40–54 years) 6-month, 1- and 2-year cumulative incidence rates of heavy menstrual bleeding were 21% (95% CI 18.2–24.8%), 30% (95% CI 25.7–33.5%) and 39% (95% CI 34.6–43.7%), respectively. Age-specific rates are given in Table 2. There was no significant statistical variation in any of these rates with age (Table 2). The overall cumulative incidence rates for heavy menstrual bleeding, with heaviness that interferes with life, were much lower, at 2.6% (95% CI 1.6–4.2%), 5.2% (95% CI 3.6–7.5%) and 7.1% (95% CI 5.1–10.0%) at the same three time points, respectively. Age-specific rates are given in Table 2.

**Table 2:** Incidence of heavy menstrual bleeding (HMB) and HMB with heaviness that interferes with life

Duration follow-up	Age (years)	Number at risk	Incident cases of HMB	6-month incidence rate of HMB (95% CI)	Incident cases of HMB with heaviness that interferes with life	6-month incidence rate of HMB with heaviness that interferes with life (95% CI)
6 months	40–44	395	87	22.0% (18.2–26.4%)	17	4.3% (2.7–6.8%)
	45–49	419	80	19.1% (15.6–23.1%)	10	2.4% (1.3–4.3%)
	50–54	218	39	17.9% (13.4–23.5%)	4	1.8% (0.7–4.6%)
				1-year cumulative incidence rate of HMB (95% CI)		1-year cumulative incidence rate of HMB with heaviness that interferes with life (95% CI)
1 year	40–44	359	108	30.1% (25.6–35.0%)	25	7.0% (4.8–10.1%)
	45–49	366	100	27.3% (23.0–32.1%)	17	4.6% (2.9–7.3%)
	50–54	202	60	29.7% (23.8–36.3%)	12	5.9% (3.4–10.1%)
				2-years cumulative incidence rate of HMB (95% CI)		2-year cumulative incidence rate of HMB with heaviness that interferes with life (95% CI)
2 years	40–44	177	76	42.9% (35.9–50.3%)	15	8.5% (5.2–13.5%)
	45–49	164	60	36.6% (29.6–44.2%)	10	6.1% (3.4–10.9%)
	50–54	92	33	35.9% (26.8–46.1%)	6	6.5% (3.0–13.5%)

### Resolution without recurrence

**Figure 1:**
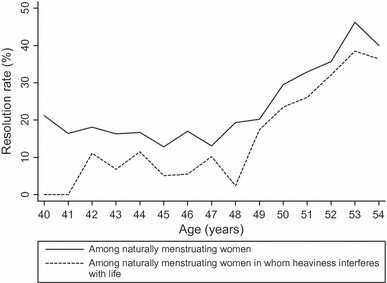
Resolution of heavy menstrual bleeding for 6 months in naturally menstruating women and in naturally menstruating women in whom heaviness interferes with life.

The overall rates of the spontaneous resolution of heavy menstrual bleeding for 6 months, 1 and 2 years, without recurrence during the time frame, were 19.4% (95% CI 17.1–21.9%), 11.8% (95% CI 9.8–14.2%) and 9.8% (95% CI 7.7–12.4%), respectively. Age-specific rates are given in Table 3. The difference between these figures calculated at the different time points is an indication of the extent to which the initial resolution (19.4%) is maintained over time (falling to 9.8% at 24 months). There is variation with age: the rate of resolution for 6 months is stable in the age range of 40–49 years, but has a sudden increase at 50 years (Figure 1); *P* < 0.001 for trend in age. The same variation with age occurs for 1- and 2-year rates of resolution without recurrence (Table 3).

**Table 3:** Rate of spontaneous resolution without recurrence of heavy menstrual bleeding (HMB)

Duration of follow-up Initial cohort	Age years	Number at risk	Cases of resolution	Rate of resolution for follow-up period (95% CI)
6 months				
HMB	40–44	670	116	17.3% (14.6–20.4%)
	45–49	670	109	16.3% (13.7–19.3%)
	50–54	302	106	35.1% (30.0–40.8%)
HMB with heaviness that interferes with life	40–44	227	16	7.0% (4.4–11.1%)
	45–49	251	20	8.0% (5.8–13.2%)
	50–54	109	32	29.4% (21.6–38.5%)
1 year				
HMB	40–44	577	55	9.5% (7.4–12.2%)
	45–49	554	51	9.2% (7.1–11.9%)
	50–54	260	71	27.3% (22.3–33.0%)
HMB with heaviness that interferes with life	40–44	184	6	3.3% (1.5–6.9%)
	45–49	206	11	5.3% (3.0–9.3%)
	50–54	89	20	22.5% (15.0–32.2%)
2 years				
HMB	40–44	258	10	3.9% (2.1–7.0%)
	45–49	248	20	8.1% (5.3–12.1%)
	50–54	126	32	25.4% (18.6–33.7%)
HMB with heaviness that interferes with life	40–44	77	2	2.6% (0.7–9.0%)
	45–49	88	7	8.0% (3.9–15.5%)
	50–54	43	9	20.9% (11.4–35.2%)

In those who also report that the heaviness interferes with their life, the overall rate of resolution without recurrence of heavy menstrual bleeding in naturally menstruating women was 11.5% (95% CI 8.6–15.1%), 8.0% (95% CI 5.5–11.7%) and 8.7% (95% CI 5.5–12.3%) for 6 months, 1 and 2 years, respectively; all three rates varied significantly (*P* < 0.001) between the three age groups (Table 3). Among the women menstruating without interference in their lives, the overall rate of resolution without recurrence of heavy menstrual bleeding in naturally menstruating women was higher at 25% (95% CI 21.3–28.0%), 42% (95% CI 38.1–46.3%) and 10.4% (95% CI 7.8–13.7%) for 6 months, 1 year and 2 years, respectively. There was a significant association of interference with life with heavy bleeding resolution without recurrence rates for 6 months and 1 year (*P* <0.001), but no significant association with resolution without recurrence for 2 years (*P* = 0.322).

There was no association of skipped periods with resolution of heavy menstrual bleeding without recurrence in the age range 40–44 years, but there was a strong association in the older age groups, with higher rates of resolution in those who reported skipped periods. In women aged 45–49 years the rate ratio, comparing rates of resolution in women with skipped periods versus those without, ranges from 2.6 (95% CI 1.9–3.6) for resolution for 6 months to 4.9 (95% CI 2.1–12) for resolution maintained without recurrence for 2 years. In women aged 50–54 years, the rate ratio varies from 2.4 (95% CI 1.6–3.5) for resolution for 6 months to 3.2 (95% CI 1.8–5.3) for resolution without recurrence for a year. The association of ‘cycle too variable to say’ with the resolution of heavy menstrual bleeding was weaker than that observed for skipped periods, but also showed age variations (Table 4).

**Table 4:** Rate ratio of skipped period and cycle too variable to say for resolution without recurrence of heavy menstrual bleeding

Initial cohort Duration of follow-up	Age years	Number of women	Resolution cases	Resolution rate (95% CI)	Rate ratio of resolution for follow-up period (95% CI)*
Skipped period					
6 months	40–44	58	12	20.7% (12.3–32.8%)	1.22 (0.70–2.05)
	45–49	185	55	29.7% (23.6–36.7%)	2.63 (1.87–3.64)
	50–54	181	83	45.9% (38.8–53.1%)	2.39 (1.58–3.47)
1 year	40–44	44	5	11.4% (5.0–24.0%)	1.23 (0.67–3.70)
	45–49	155	31	20.0% (14.5–27.0%)	4.08 (2.36–6.84)
	50–54	156	59	37.8% (30.6–45.6%)	3.15 (1.78–5.33)
2 years	40–44	19	0	0% (–)	0
	45–49	68	13	19.1% (11.5–30.0%)	4.90 (2.05–11.81)
	50–54	89	28	31.5% (22.8–41.7%)	2.92 (1.10–7.71)
Cycle too variable to say					
6 months	40–44	22	4	18.2% (7.3–38.5%)	1.46 (0.43–2.64)
	45–49	88	28	31.8% (23.0–42.1%)	2.22 (1.58–3.29)
	50–54	70	36	51.4% (40.0–62.8%)	1.70 (1.27–2.33)
1 year	40–44	18	1	5.6% (1.0–25.8%)	0.58 (0.08–3.96)
	45–49	72	12	16.7% (9.8–26.9%)	2.06 (1.13–3.75)
	50–54	61	28	45.9% (34.0–58.3%)	2.23 (1.47–3.19)
2 years	40–44	12	1	8.3% (1.5–35.4%)	2.24 (0.31–16.6)
	45–49	36	4	11.1% (4.4–25.3%)	1.48 (0.52–4.15)
	50–54	29	13	44.8% (28.4–62.5%)	2.29 (1.29–4.05)

* Ratio of resolution in those with skipped period/cycle too variable to say to those without skipped period/cycle not too variable to say.

Stratified analysis with key modifying factors (basal metabolic index, race, current smoking status and work status) failed to reveal statistically significant differences or associations with resolution.

### Health care use

The annual cumulative incidence of consultation with a doctor and/or nurse during the previous 6 months about the heaviness of periods in naturally menstruating women was 6.9 per 100 per year. The rate for those with heavy menstrual bleeding was 9.7 per 100 per year, and for those with heavy menstrual bleeding in whom the heaviness interferes with life the annual rate was 17 per 100 per year.

## Discussion

We have found a high prevalence, incidence and spontaneous rate of resolution of heavy menstrual bleeding in naturally menstruating women during the perimenopausal years. Approximately two-thirds of naturally menstruating women aged 40–54 years reported heavy menstrual bleeding in the previous 6 months.

Among the one-third of naturally menstruating women aged 40–54 years who have not had heavy menstrual bleeding in the past 6 months, at least one-third will develop it at some time in the next 2 years.

The rates of resolution of heavy menstrual bleeding are also high at these ages. Overall, a fifth of women with heavy menstrual bleeding will experience its spontaneous resolution for the next 6 months, but in only half will resolution without recurrence be maintained for 2 years. There is considerable variation with age in the rates of resolution and maintenance of the symptom-free state, with those in the younger age groups having lower rates. Heavy menstrual bleeding that interferes with life has a lower prevalence and incidence, but resolution rates are also lower in this group.

There is a strong association between the subsequent spontaneous resolution of heavy menstrual bleeding and the reporting of skipped periods in the age range of 45–54 years: women with skipped periods are between two and five times more likely to experience the spontaneous resolution of heavy menstrual bleeding, compared with those who do not report the symptom. The reporting of a cycle that is ‘too variable to say’ is generally associated with the subsequent spontaneous resolution of heavy menstrual bleeding in this age group, but the association is weaker than that for skipped periods.

A strength of the study is that it used the whole adult female population aged 40–54 years registered with urban and rural group general practices, covering a range of affluence and deprivation. The response rate to the baseline questionnaire of 63% was consistent with previously published studies of this type. Responders were slightly older than non-responders, but given the lack of age-specific effects on our main measures of occurrence, this difference is unlikely to have materially affected the population estimates reported here. The response rate to follow-up questionnaires was high, at over 80%.

The study was based on the self-reporting of heavy menstrual bleeding. This may be subject to recall and observer bias. The size of this effect in this study is not known. Objective assessments using diaries or charts tend to give a lower prevalence than subjective assessment, but even this method is not free from bias, as the majority are not completed at the allotted time.^22^ The subjective methodology used in this study is comparable with clinical practice in primary care in the UK, where it is rare for a woman to present with a completed menstrual chart. National guidelines for the management of heavy menstrual bleeding do not recommend the use of menstrual charts.^10^ The time frame used of 6 months creates an imprecision in the rates of resolution, as the duration of resolution for an individual may be up to 6 months longer than reported here, depending on when the last event occurred in the previous 6 months. This will result in an underestimate of the rates of resolution, compared with studies using an objective measurement that locates the event more precisely in time. The questionnaire as a whole had not been validated in previous studies, the performance characteristics of the individual questions are unpublished, and the questionnaire requires a degree of literacy to complete. The effect of this potential source of bias is unknown.

The participants in this study were a community population, and therefore differ from those consulting primary care. It is known that women who consult primary care with increased vaginal bleeding are different from those in the community who report the same symptom, yet do not consult, and that the main driver for consultation with primary care is interference with life.^8^ Rates of spontaneous resolution of heavy menstrual bleeding in women who report that it interferes with their life were lower than the figures for all women with heavy menstrual bleeding. Although this was not a consulting population, the women in our study with symptoms that interfere with life will be disproportionately represented in a consulting population, as evidenced by our finding that the annual cumulative incidence of consultation about heaviness of periods overall was 6.9%, compared with 17% among women who also reported that the heaviness interfered with life. Applying the rates of resolution observed here directly to consulting populations should be undertaken with caution. Watchful waiting may be an acceptable line of management in primary care, as the majority of women who consult secondary care with heavy menstrual bleeding have no detectable pathology, and, although research on primary care populations is lacking,^10^ the rate of significant pathology in primary care is likely to be even lower. Using the rates of spontaneous resolution of heavy menstrual bleeding, in conjunction with interference with life, and the symptoms of skipped periods and cycle ‘too variable to say’, will allow a more informed decision about undertaking treatment or not.

The study failed to show statistically significant differences or associations with key modifying factors. The study may have been underpowered to reveal such associations, as the sample size was not calculated with this as the primary outcome.

An earlier study on menstruating women gave the prevalence of heavy menstrual bleeding as 53% of women aged 45–54 years. The rate in our study was higher, but unlike the earlier study we excluded women using female hormones, treatments for heavy menstrual bleeding and endometrial ablation, all of which are known to reduce menstrual loss. The 12-month cumulative incidence of heavy menstrual bleeding in the age group 45–54 years of 27% in the earlier study is comparable with that in our study, with a rate of 28%. There are difficulties in comparing studies on heavy menstrual bleeding because of differences in the definition of the symptom (objective or subjective assessment, for example) and characteristics of the study population (all women or menstruating women, age bands and use of contraception, for example). A comparable community study to ours gave a prevalence of 39% of menstruating women aged 35–59 years self-reporting heavy periods in the previous 6 months,^3^ whereas a study using an objective assessment of menstrual loss gave the prevalence of heavy menstrual bleeding of 15% of menstruating women aged 40 years and older.^1^

The prevalence of skipped periods in this study is consistent with the observation that this symptom is common before the menopause.^23^ The association between the spontaneous resolution of heavy menstrual bleeding and skipped periods, or cycle ‘too variable to say’, is consistent with the postulate that these symptoms are predictive of the menopause.^18^

## Conclusions

This study provides the first published estimates of the likelihood that perimenopausal women in the community will experience a resolution of heavy menstrual bleeding with no treatment. This information is of importance for women themselves, for decisions in clinical consultations, and for those devising and implementing interventions, and also provides a population context for decisions about the more severe subgroups that seek care.

## Disclosure of interests

All authors declare that they have no interests to declare.

## Contribution to authorship

MS produced the original idea, and MS, KJ and PC designed the study. MB and MS analysed the data, and all authors wrote up the study.

## Details of ethical approval

Approval was obtained from Staffordshire Research Ethics Committee (06/Q2602/38).

## Funding

Funding was received from NHS(E) West Midlands R&D office and North Staffordshire Primary Care R&D Consortium.
